# Inhibition of 15‐hydroxyprostaglandin dehydrogenase protects neurons from ferroptosis in ischemic stroke

**DOI:** 10.1002/mco2.452

**Published:** 2024-01-07

**Authors:** Yunfei Xu, Kexin Li, Yao Zhao, Lin Zhou, Nina He, Haoduo Qiao, Qing Xu, Huali Zhang, Ying Liu, Jie Zhao

**Affiliations:** ^1^ Department of Pathophysiology School of Basic Medical Sciences Central South University Changsha Hunan China; ^2^ Department of Neurosurgery Xiangya Hospital Central South University Changsha Hunan China; ^3^ Sepsis Translational Medicine Key Lab of Hunan Province Changsha Hunan China; ^4^ National Medicine Functional Experimental Teaching Center Central South University Changsha Hunan China; ^5^ Postdoctoral Research Station of Biology School of Basic Medical Science Central South University Changsha Hunan China

**Keywords:** 15‐PGDH, ferroptosis, GPX4, ischemic stroke, PGE2

## Abstract

Ischemic stroke is an acute serious cerebrovascular disease with high mortality and disability. Ferroptosis is an important regulated cell death (RCD) in ischemic stroke. 15‐Hydroxyprostaglandin dehydrogenase (15‐PGDH), a degrading enzyme of prostaglandin E2 (PGE2), is shown to regulate RCD such as autophagy and apoptosis. The study aimed to determine whether 15‐PGDH regulates ferroptosis and ischemic stroke, and further the exact mechanism. We demonstrated that overexpression of 15‐PGDH in the brain tissues or primary cultured neurons significantly aggravated cerebral injury and neural ferroptosis in ischemic stroke. While inhibition of 15‐PGDH significantly protected against cerebral injury and neural ferroptosis, which benefits arise from the activation of the PGE2/PGE2 receptor 4 (EP4) axis. While the impact of 15‐PGDH was abolished with glutathione peroxidase 4 (GPX4) deficiency. Then, 15‐PGDH inhibitor was found to promote the activation of cAMP‐response element‐binding protein (CREB) and nuclear factor kappa‐B (NF‐κB) via the PGE2/EP4 axis, subsequently transcriptionally upregulate the expression of GPX4. In summary, our study indicates that inhibition of 15‐PGDH promotes the activation PGE2/EP4 axis, subsequently transcriptionally upregulates the expression of GPX4 via CREB and NF‐κB, and then protects neurons from ferroptosis and alleviates the ischemic stroke. Therefore, 15‐PGDH may be a potential therapeutic target for ischemic stroke.

## Graphical abstract

Ischemic stroke simultaneously induces the massive release of PGE2 and ferroptosis of neurons. While the PGE2 degrading enzyme 15‐PGDH degrades PGE2 and subsequently reduces the level of EP4, a receptor of PGE2, which can transcriptionally reduce GPX4 level to promote ferroptosis through CREB and NF‐κB, finally exacerbates ischemic stroke

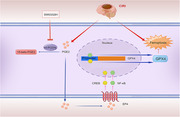

## 1 INTRODUCTION

Stroke is a severe acute cerebrovascular disease. As the second‐leading cause of death and a leading cause of long‐term disability, stroke imposes a huge burden on global human health.^1^ Among all new strokes in 2019, ischemic stroke constituted 62.4%. Ischemic stroke is a focal cerebral infarct caused by cerebrovascular blockage.^2^ The immediate consequence of cerebrovascular blockage is an inadequate supply of oxygen and glucose in the ischemic region, which then triggers multiple pathophysiologies such as inflammation, oxidative stress, and excitotoxicity, eventually leading to cell death.^3^ High systolic blood pressure, high body mass index, high fasting plasma glucose, ambient particulate matter pollution, and smoking are leading risk factors for stroke. Intravenous thrombolysis by tissue plasminogen activator or urokinase is the best treatment to restore blood perfusion of ischemic brain tissue, while its golden therapeutic window is strictly limited to six hours.^4^ Developing new therapeutic strategies for ischemic stroke is of great significance.

Ferroptosis is a new form of regulated cell death (RCD) proposed in 2012, differing from other classical RCD such as apoptosis and autophagy.^5^ Ferroptosis is characterized by iron‐dependent lipid reactive oxygen species (L‐ROS) overproduction, with shrinkable mitochondrial and increased mitochondrial membrane density, ruptured or vanishing of mitochondria crista, while normal nuclei.^6^ The depletion of glutathione (GSH) or the inactivation of glutathione peroxidase 4 (GPX4) is an underlying biochemical mechanism of ferroptosis.^7^ Due to the strong antioxidant capacity of GSH against ferroptosis, restoring the level of GSH or GPX4 is one of the important strategies to inhibit ferroptosis. Recent studies revealed that ferroptosis is implicated in the occurrence and development of several diseases, such as ischemia–reperfusion injury (IRI), cancer, acute kidney injury, diabetes, neurodegenerative diseases, stroke, SARS‐CoV‐2 infection, and brain trauma.^8^, ^9^, ^10^ However, the research on the relevance of ferroptosis in ischemic stroke is still superficial.^11^ To further explore the role of ferroptosis in ischemic stroke is of great significance for the treatment of ischemic stroke.

15‐Hydroxyprostaglandin dehydrogenase (15‐PGDH) is the degrading enzyme of prostaglandin E2 (PGE2) that converts PGE2 to inactivate 15‐keto‐PGE2.^12^ After degrading PGE2, 15‐PGDH plays important roles in a variety of pathophysiology by activating downstream signaling pathways via PGE2 receptors (EP1–EP4). Partly by reducing the level of 15‐PGDH, SARS‐CoV‐2 induces PGE2 generation and secretion in infected lung epithelial cells.^13^ 15‐PGDH inhibition by inhibitor SW033291 or knockout upregulates PGE2 levels in the colon, liver, and bone marrow, then potentiates tissue repair and regeneration via EP2 and EP4.^14^ In aged murine skeletal muscles, elevated 15‐PGDH leads to the reduction of PGE2 concentrations. Inhibition of 15‐PGDH, either by a short hairpin RNA or SW033291, rejuvenates aged muscle mass and strength, which is mediated by PGE2 and EP4.^12^ However, 15‐PGDH has rarely been reported in ischemic stroke. Our previous study has confirmed that PGE2 inhibits ischemic stroke‐induced ferroptosis, and EP3 and EP4 are sensitive to ferroptosis, while we have not delved into the exact mechanisms.^15^ Hence, we aim to determine whether 15‐PGDH regulates ischemic stroke‐induced ferroptosis through the PGE2–EP pathway, and to further explore its molecular mechanism.

In the present study, we hypothesize that 15‐PGDH downregulates the levels of PGE2, then contributes the ferroptosis induced by ischemic stroke via some EPs. To test the hypothesis, we determined the effect of 15‐PGDH on ischemic stroke and ferroptosis, and investigated which EPs mediating this process and the regulated target molecules of ferroptosis, finally revealed the transcriptional regulation mechanism of the EPs on the target molecules. Our results confirm that 15‐PGDH exacerbates ischemic stroke by promoting ferroptosis. In this process, 15‐PGDH promotes the activation of cAMP‐response element‐binding protein (CREB) and nuclear factor kappa‐B (NF‐κB) by inhibiting the PGE2/EP4 axis, and transcriptionally reduces GPX4 level, ultimately promoting ferroptosis.

## 2 RESULTS

### 2.1 15‐PGDH aggravates ischemic stroke in vivo and in vitro

**FIGURE 1:**
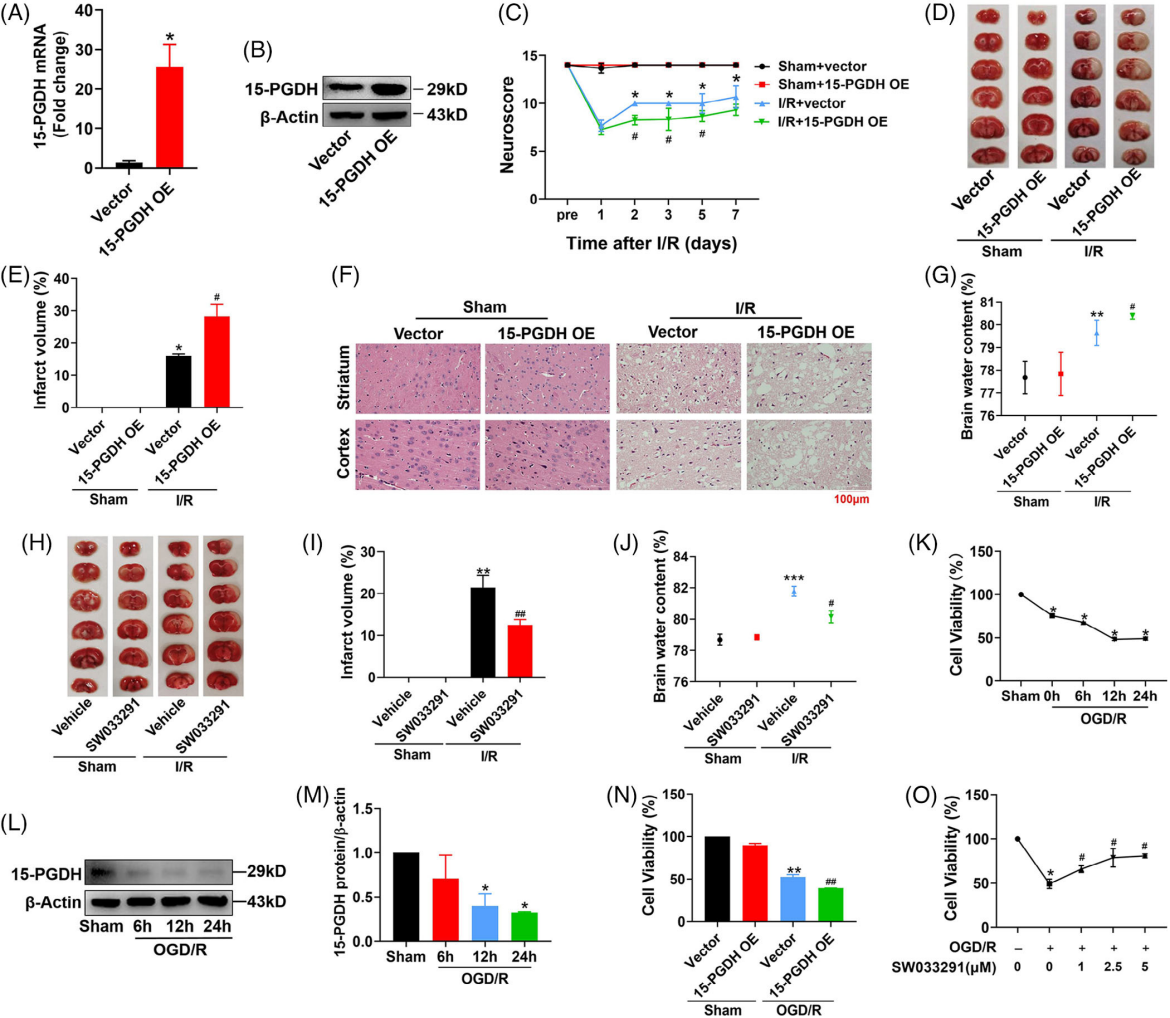
15‐PGDH aggravates ischemic stroke in vivo and in vitro. (A and B) mRNA and protein expression levels of 15‐PGDH after overexpression AAV9 infected for 2 weeks. (C) Neurological deficits were assessed before ischemic stroke and 1, 2, 3, 5, and 7 days after ischemic stroke. (D and E) 15‐PGDH overexpression exacerbated cerebral infarction in rats as determined by TTC staining. (F) 15‐PGDH overexpression led to more severe structural destruction of brain tissue in ischemic stroke. (G) 15‐PGDH overexpression increased brain water content in MCAO rats, scale bars: 50 μm. (H) 15‐PGDH inhibition decreased brain water content in MCAO rats. (I and J) 15‐PGDH inhibition alleviated cerebral infarction in rats as determined by TTC staining. (K) Cell viability of rat primary cortical neurons in an OGD/R model was determined by CCK‐8 assay. (L and M) Protein levels of 15‐PGDH in an OGD/R model were detected by western blot. (N) Cell viability of rat primary cortical neurons after transfected with 15‐PGDH overexpression plasmid for 48 h in an OGD/R model was determined by CCK‐8 assay. (O) Cell viability of rat primary cortical neurons after treatment with different concentrations of SW033291 for 12 h in an OGD/R model was determined by CCK‐8 assay. Data are shown as the mean ± SD, *n* = 6 in (A–J), *n* = 3 in (K–O). *p < 0.05, **p < 0.01, ***p < 0.001 VS Sham+Vector group or Sham+Vehicle group; #*p* < 0.05, ##*p* < 0.01, VS I/R (OGD/R) +Vector group or I/R (OGD/R) +Vehicle group.

As our previous study has demonstrated that stroke induces a reduction of 15‐PGDH in a rat middle cerebral artery occlusion (MCAO) model,^16^ we first tested the effects of overexpression 15‐PGDH on ischemic stroke in vivo. The right lateral ventricle was stereotaxically injected with 15‐PGDH overexpression adeno‐associated virus (AAV9) and infected for 2 weeks. AAV successfully induced a significant upregulation of 15‐PGDH expression (Figures 1A and B). Then, we established an MCAO rat model and determined the effects of overexpression 15‐PGDH on ischemic stroke. Neurological deficits analysis was performed before ischemic stroke and 1, 2, 3, 5, and 7 days after ischemic stroke, and 15‐PGDH overexpression was observed to exacerbate neurological impairments on 2, 3, 5, and 7 days after ischemic stroke (Figure 1C). Notably, increased infarct volume was induced by 15‐PGDH overexpression (Figures 1D and E). Besides, hematoxylin–eosin (HE) staining showed that 15‐PGDH overexpression promoted the destruction of brain structures (Figure 1F). Higher brain water content indicates more severe brain edema after 15‐PGDH overexpression (Figure 1G). In a word, 15‐PGDH overexpression was confirmed to aggravate ischemic stroke.

We further verified the function of 15‐PGDH inhibition in a rat MCAO model with SW033291 to inhibit the enzyme activity of 15‐PGDH.^14^ Two hours before the establishment of MCAO model, 0.075 mg/kg SW033291 was stereotaxically injected into the right lateral ventricle. After inhibition of 15‐PGDH, the rats exhibited a decreased infarct volume and lower brain water content (Figures 1H–J). These results suggested that 15‐PGDH inhibition alleviated ischemic stroke.

In vitro, primary cortical neurons of rats were extracted to determine the effects of 15‐PGDH on ischemic stroke. As IC50 of oxygen‐glucose deprivation (OGD)/R model reached at 12 h after reperfusion, this time point was selected for subsequent experiments (Figure 1K). In OGD/R model, 15‐PGDH was also decreased, which was consistent with in vivo (Figures 1L and M). Then, the 15‐PGDH overexpression plasmid was transfected for 48 h, inducing a decreased cell activity in OGD/R model (Figure 1N). Conversely, cell activity increased after 15‐PGDH was inhibited by SW033291 (Figure 1O). These results were consistent with those in vivo.

### 2.2 15‐PGDH promotes ischemic stroke‐induced ferroptosis

**FIGURE 2:**
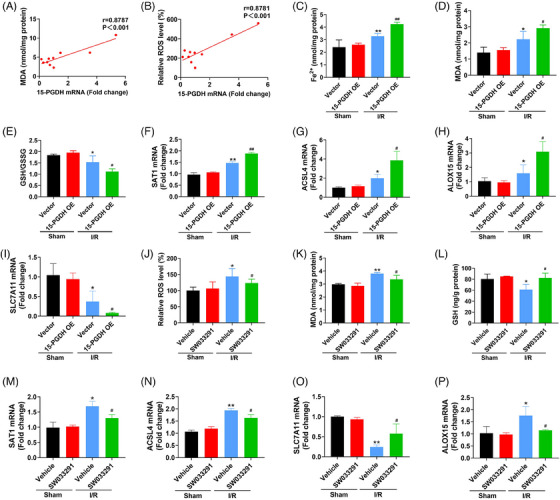
15‐PGDH promotes ischemic stroke‐induced ferroptosis. (A) Correlation analysis of 15‐PGDH mRNA level and MDA concentration in brain tissue samples from patients with brain injury. (B) Correlation analysis of 15‐PGDH mRNA level and relative ROS level in brain tissue samples from patients with brain injury. (C) Fe^2+^ content was measured by colorimetry after 15‐PGDH overexpression AAV9 infected for 2 weeks in an MCAO model. (D) MDA content was measured by colorimetry after 15‐PGDH overexpression AAV9 infected for 2 weeks in an MCAO model. (E) The ratio of GSH/GSSG was measured after 15‐PGDH overexpression AAV9 infected for 2 weeks in an MCAO model. (F–I) The mRNA levels of SAT1, ACSL4, SLC7A11, and ALOX15 were measured by RT‐qPCR after 15‐PGDH overexpression AAV9 infected for 2 weeks in an MCAO model. (J) Relative ROS level was measured by colorimetry after SW033291 treatment in an MCAO model. (K) MDA content was measured by colorimetry after g SW033291 treatment in an MCAO model. (L) GSH content was measured after SW033291 treatment in an MCAO model. M‐P The mRNA levels of SAT1, ACSL4, SLC7A11, and ALOX15 were measured by RT‐qPCR after SW033291 treatment in an MCAO model. Data are shown as the mean ± SD, *n* = 10 in (A and B), *n* = 6 in (C–P). *p < 0.05, **p < 0.01, vs sham + vector group or sham + vehicle group; #*p* < 0.05, ##*p* < 0.01, vs I/R + vector group or I/R + vehicle group.

Our previous study has confirmed the presentation of ferroptosis in ischemic stroke.^15^ In addition, the results above indicated that 15‐PGDH aggravated ischemic stroke in vivo. Therefore, we examined the effects of 15‐PGDH on ferroptosis to determine whether 15‐PGDH regulates ischemic stroke through ferroptosis. As lipid peroxidation is the core process of ferroptosis, we first determined the correlation between 15‐PGDH and lipid peroxidation. After collecting brain tissues from patients with brain injury, the levels of 15‐PGDH, ROS, and malondialdehyde (MDA) were measured. The results showed that 15‐PGDH was positively correlated with ROS and MDA (Figures 2A and B), indicating that 15‐PGDH may also be positively correlated with lipid peroxidation, even ferroptosis. To determine the exact effects of 15‐PGDH on ischemic stroke‐induced ferroptosis, 15‐PGDH overexpression AAV was infected for two weeks. Increased Fe^2+^ (Figure 2C), MDA (Figure 2D), reduced ratio of GSH/oxidized glutathione (GSSG) (Figure 2E), increased spermidine/spermine N1‐acetyltransferase 1 (SAT1) (Figure 2F), acyl‐CoA synthetase long‐chain family member 4 (ACSL4) (Figure 2G), arachidonate lipoxygenase 15 (ALOX15) (Figure 2H), and reduced subunit solute carrier family 7 member 11 (SLC7A11) (Figure 2I) were observed after 15‐PGDH overexpression. These results indicate that 15‐PGDH overexpression promotes ischemic stroke‐induced ferroptosis. Conversely, inhibition of 15‐PGDH by SW033291 induced decreased ROS, MDA, SAT1 and ACSL4, ALOX15 and increased GSH and SLC7A11 (Figures 2J–P). 15‐PGDH inhibition suppressed ischemic stroke‐induced ferroptosis.

### 2.3 15‐PGDH promotes OGD/R‐induced neuronal ferroptosis

**FIGURE 3:**
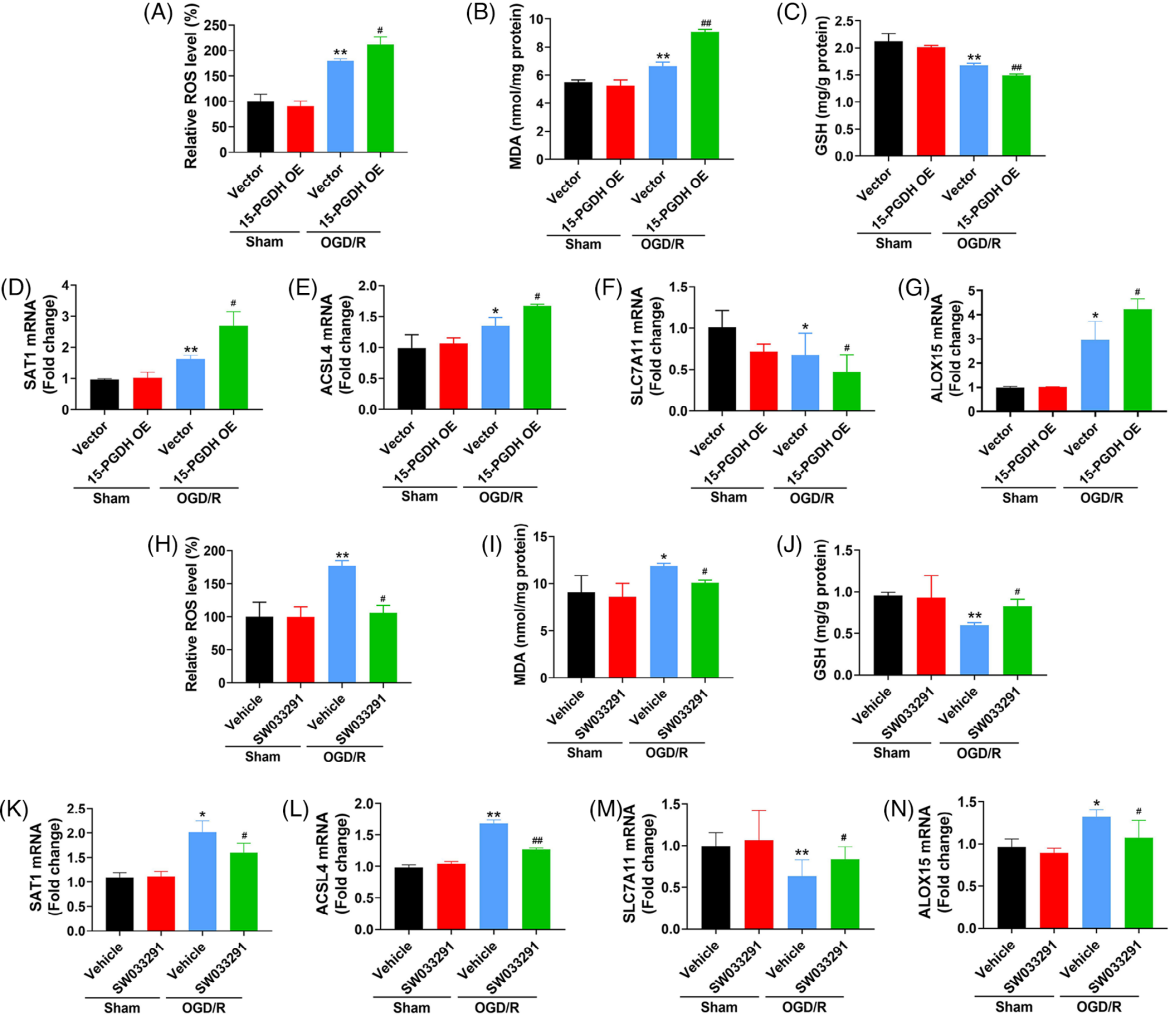
15‐PGDH promotes OGD/R‐induced neuronal ferroptosis. (A) Relative ROS level was measured by colorimetry after transfected with 15‐PGDH overexpression plasmid for 48 h in a OGD/R model. (B) MDA content was measured by colorimetry after transfected with 15‐PGDH overexpression plasmid for 48 h in a OGD/R model. (C) GSH content was measured by colorimetry after transfected with 15‐PGDH overexpression plasmid for 48 h in a OGD/R model. (D–G) The mRNA levels of SAT1, ACSL4, SLC7A11, and ALOX15 were measured by RT‐qPCR after transfected with 15‐PGDH overexpression plasmid for 48 h in a OGD/R model. (H) Relative ROS level was measured by colorimetry after SW033291 treatment in a OGD/R model. (I) MDA content was measured by colorimetry after SW033291 treatment in a OGD/R model. (J) GSH content was measured by colorimetry after SW033291 treatment in a OGD/R model. (K–N) The mRNA levels of SAT1, ACSL4, SLC7A11, and ALOX15 were measured by RT‐qPCR after SW033291 treatment in a OGD/R model. Data are shown as the mean ± SD, *n* = 3 *p < 0.05, **p < 0.01, vs sham + vector group or sham + vehicle group; #*p* < 0.05, ##*p* < 0.01, vs OGD/R + vector group or OGD/R + vehicle group.

As the above results have investigated the effects of 15‐PGDH on ischemic stroke‐induced ferroptosis in vivo, we further assessed the effects of 15‐PGDH on OGD/R‐induced neuronal ferroptosis in vitro. We extracted primary cortical neurons from rats and transfected 15‐PGDH overexpression plasmid for 48 h. In OGD/R model, elevated ROS, MDA, SAT1 and ACSL4, ALOX15, and decreased GSH, SLC7A11 were presented after 15‐PGDH overexpression, indicating 15‐PGDH promotes OGD/R‐induced neuronal ferroptosis (Figures 3A–G). On the contrary, after 15‐PGDH inhibited by SW033291, reduced ROS, MDA, SAT1 and ACSL4, ALOX15, and raised GSH and SLC7A11 were obviously observed, suggesting 15‐PGDH inhibition attenuated OGD/R‐induced neuronal ferroptosis (Figures 3H–N). These results were consistent with those results in vivo.

### 2.4 GPX4 is involved in the regulation of 15‐PGDH on ischemic stroke and ferroptosis toxicity

**FIGURE 4:**
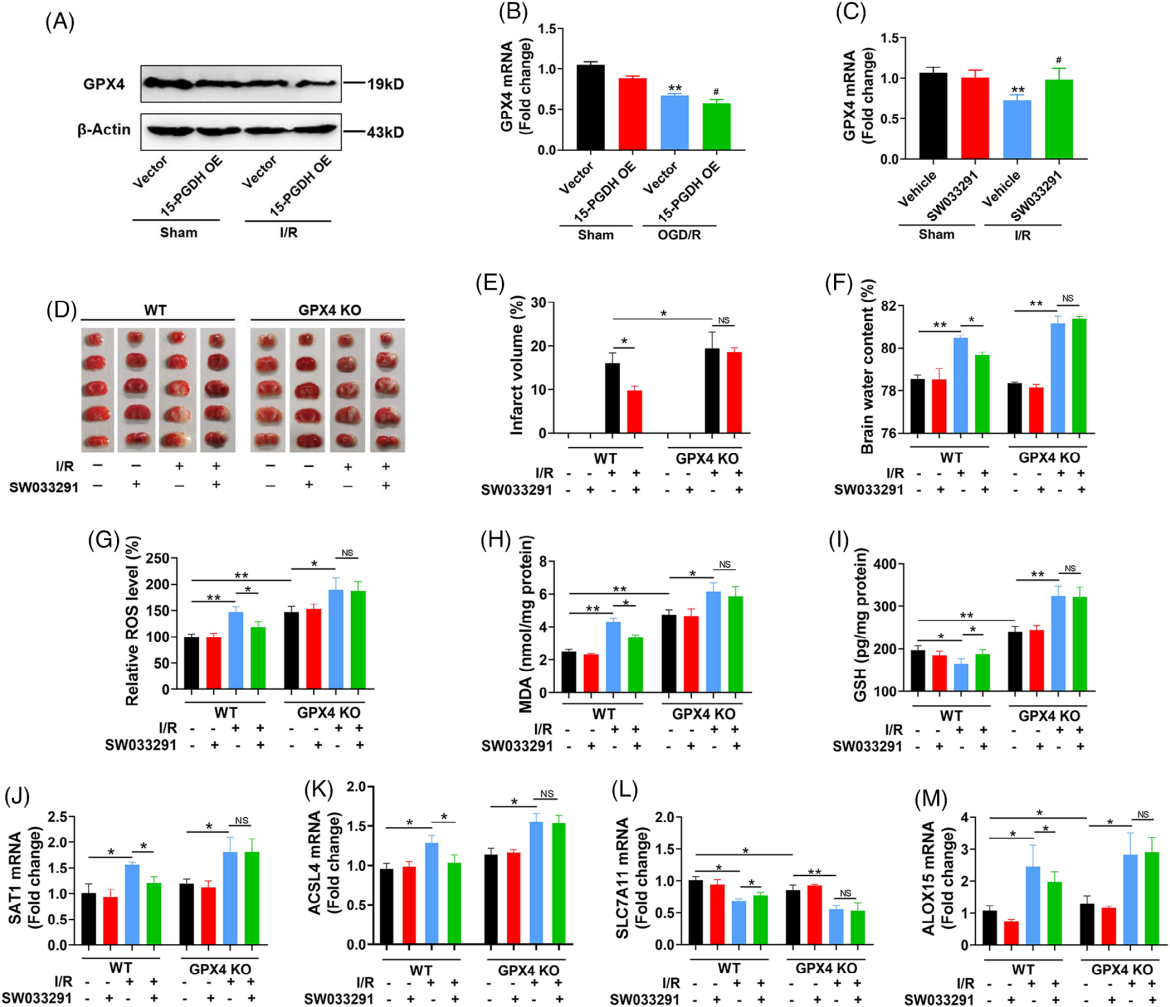
GPX4 is involved in the regulation of 15‐PGDH on ischemic stroke and ferroptosis toxicity. (A) Protein expression level of GPX4 after 15‐PGDH overexpression AAV9 infected for 2 weeks in an MCAO model. (B) mRNA expression levels of GPX4 after SW033291 treatment in an MCAO model. (C) mRNA expression levels of GPX4 after transfected with 15‐PGDH overexpression plasmid for 48 h in an OGD/R model. (D and E) TTC staining exhibiting cerebral infarction after SW033291 treatment with or without GPX4 deficiency in an MCAO model. (F) Brain water content was determined after SW033291 treatment with or without GPX4 deficiency in an MCAO model. (G) Relative ROS level was measured by colorimetry after SW033291 treatment with or without GPX4 deficiency in an MCAO model. (H) MDA content was measured by colorimetry after SW033291 treatment with or without GPX4 deficiency in an MCAO model. (I) GSH content was measured by colorimetry after SW033291 treatment with or without GPX4 deficiency in an MCAO model. (J–M) The mRNA levels of SAT1, ACSL4, SLC7A11 and ALOX15 were measured by RT‐qPCR after SW033291 treatment with or without GPX4 deficiency in an MCAO model. Data are shown as the mean ± SD, *n* = 6 in (A and B) and (E–M), *n* = 3 in (C). *p < 0.05, **p < 0.01, VS Sham+Vector group or Sham+Vehicle group; #*p* < 0.05, ##*p* < 0.01, VS I/R (OGD/R) +Vector group or I/R (OGD/R) +Vehicle group.

The above results have revealed that 15‐PGDH aggravated ischemic stroke and ischemic stroke‐induced ferroptosis. We further determined whether 15‐PGDH aggravated ischemic stroke by exacerbating ferroptosis and explored the exact target molecule in ferroptosis regulated by 15‐PGDH. GSH is an important antioxidant in ferroptosis, which depends on the catalysis of GPX4. Altering the level of GPX4 is an effective strategy to intervene in ferroptosis.^17^ Therefore, we investigated whether the regulation of 15‐PGDH on ischemic stroke and ferroptosis was dependent on GPX4. We observed that both in vivo and in vitro, 15‐PGDH overexpression downregulated the level of GPX4 (Figures 4A and B), while 15‐PGDH inhibition upregulated the level of GPX4 (Figure 4C), indicating that 15‐PGDH may regulate ferroptosis by altering GPX4 level.

To confirm the crucial role of GPX4 in this process, we constructed GPX4 conditional knockout (CKO) mice to explore whether 15‐PGDH inhibitor SW033291 can still ameliorate ischemic stroke and ferroptosis with GPX4 deficiency. At 0 and 12 h after mouse MCAO model establishment, SW033291 was intraperitoneally injected into the mice at a dose of 10 μg/g body weight. In wild type mice, SW033291 markedly attenuated MCAO‐induced cerebral infarction and edema. While in GPX4 CKO mice, the protective effect of SW033291 on ischemic stroke was not observed (Figures 4D–F). These results confirmed that the effects of 15‐PGDH on ischemic stroke was dependent on GPX4. Meanwhile, in wild type mice, SW033291 induced the reduction of ROS, MDA, SAT1 and ACSL4, ALOX15 and the rise of GSH and SLC7A11, while these effects were eliminated with GPX4 deficiency (Figures 4G–M). The above results revealed that GPX4 mediated the regulation of 15‐PGDH on ischemic stroke and ferroptosis.

### 2.5 15‐PGDH regulates ischemic stroke through the PGE2/EP4 axis

**FIGURE 5:**
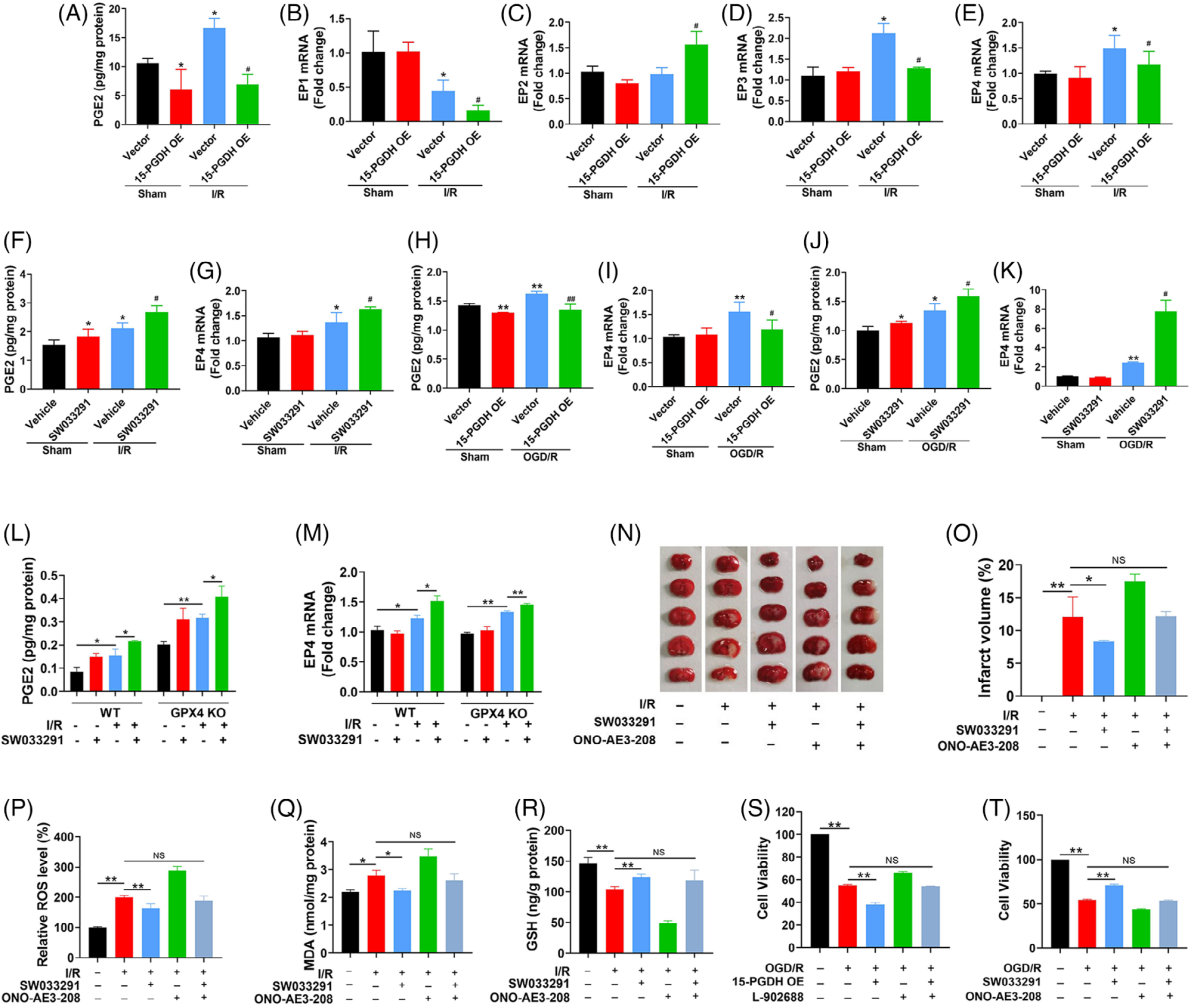
15‐PGDH regulates ischemic stroke through the PGE2/EP4 axis. (A) PGE2 concentration was detected after 15‐PGDH overexpression AAV9 infected for 2 weeks in an MCAO model. (B–E) The mRNA levels of EP1, EP2, EP3, and EP4 were measured by RT‐qPCR after 15‐PGDH overexpression AAV9 infected for 2 weeks in an MCAO model. (F) PGE2 concentration was detected after SW033291 treatment in an MCAO model. (G) The mRNA levels of EP4 were measured by RT‐qPCR after SW033291 treatment in an MCAO model. (H) PGE2 concentration was detected after transfected with 15‐PGDH overexpression plasmid for 48 h in a OGD/R model. (I) The mRNA levels of EP4 were measured by RT‐qPCR after transfected with 15‐PGDH overexpression plasmid for 48 h in a OGD/R model. (J) PGE2 concentration was detected after SW033291 treatment in a OGD/R model. (K) The mRNA levels of EP4 were measured by RT‐qPCR after SW033291 treatment in a OGD/R model. (L) PGE2 concentration was detected after SW033291 treatment with or without GPX4 deficiency in an MCAO model. (M) The mRNA levels of EP4 were measured by RT‐qPCR after SW033291 treatment with or without GPX4 deficiency in an MCAO model. (N and O) TTC staining exhibiting cerebral infarction after SW033291 and ONO‐AE3‐208 treatment in an MCAO model. (P) Relative ROS level was measured by colorimetry after SW033291 and ONO‐AE3‐208 treatment in an MCAO model. (Q) MDA content was measured by colorimetry after SW033291 and ONO‐AE3‐208 treatment in an MCAO model. (R) GSH content was measured by colorimetry after SW033291 and ONO‐AE3‐208 treatment in an MCAO model. (S) Cell viability was determined after 15‐PGDH overexpression plasmid for 48 h with or without EP4 agonist L‐902688. (T) Cell viability was determined after SW033291 treatment with or without EP4 antagonist ONO‐AE3‐208. Data are shown as the mean ± SD, *n* = 6 in (A–G) and (L–R), *n* = 3 in (H–K) and (S and T). *p < 0.05, **p < 0.01, vs sham + vector group or sham + vehicle group; #*p* < 0.05, ##*p* < 0.01, vs I/R (OGD/R) + vector group or I/R (OGD/R) + vehicle group.

PGE2 is known to bind to specific EPs to activate multiple downstream signaling pathways, such as PKA/CREB, GSK3β/β‐catenin, PI3K/AKT/mTOR, and NF‐κB, to perform different biological functions.^18^, ^19^, ^20^, ^21^, ^22^, ^23^ As the sole degradation enzyme of PGE2, 15‐PGDH works via PGE2/EPs axis in a variety of pathophysiology. Therefore, we speculated that the effect of 15‐PGDH on ischemic stroke was through the PGE2/EPs axis, and then identified the crucial EP. In vivo, 15‐PGDH overexpression led to a reduction of PGE2 (Figure 5A), as well as the decrease of EP1, EP2, EP4 and the increase of EP3 (Figure 5B–E). Previous reports have shown that the decrease of EP1, EP2 and the increase of EP3 were neuroprotective in stroke, and only the decrease of EP4 aggravates ischemic stroke, which is consist with the effects of 15‐PGDH overexpression on ischemic stroke.^24^ Therefore, we preliminarily speculated that the effects of 15‐PGDH on ischemic stroke was mediated by the PGE2/EP4 axis. Conversely, 15‐PGDH inhibition by SW033291 promoted the PGE2/EP4 axis in vivo (Figures 5F and G). Meanwhile, the effects of 15‐PGDH on PGE2/EP4 axis were also observed in vitro (Figures 5H–K). Notably, SW033291 was able to promote the PGE2/EP4 axis with GPX4 deficiency (Figures 5L and M), indicating that the PGE2/EP4 axis acts upstream of GPX4. To confirm the indispensable role of EP4 in this process in vivo, 15‐PGDH inhibitor SW033291 and EP4 antagonist ONO‐AE3‐208 were simultaneously administrated in a MCAO mouse model. The ability of SW033291 to reduce infarct volume, ROS, MDA and increase GSH was abolished by ONO‐AE3‐208 (Figures 5N–R). In vitro, 10 μmol/L EP4 agonist L‐902688 or 10 μmol/L antagonist ONO‐AE3‐208 was respectively used after 15‐PGDH overexpression or inhibition. The results showed that after EP4 levels were reversed by its agonist or antagonist, the regulation of 15‐PGDH on cell activity in a OGD/R model was eliminated (Figures 5S and T). These findings demonstrated that PGE2/EP4 axis mediates the regulation of 15‐PGDH on ischemic stroke.

### 2.6 15‐PGDH/PGE2/EP4 transcriptionally regulates GPX4 expression through CREB and NF‐κB

**FIGURE 6:**
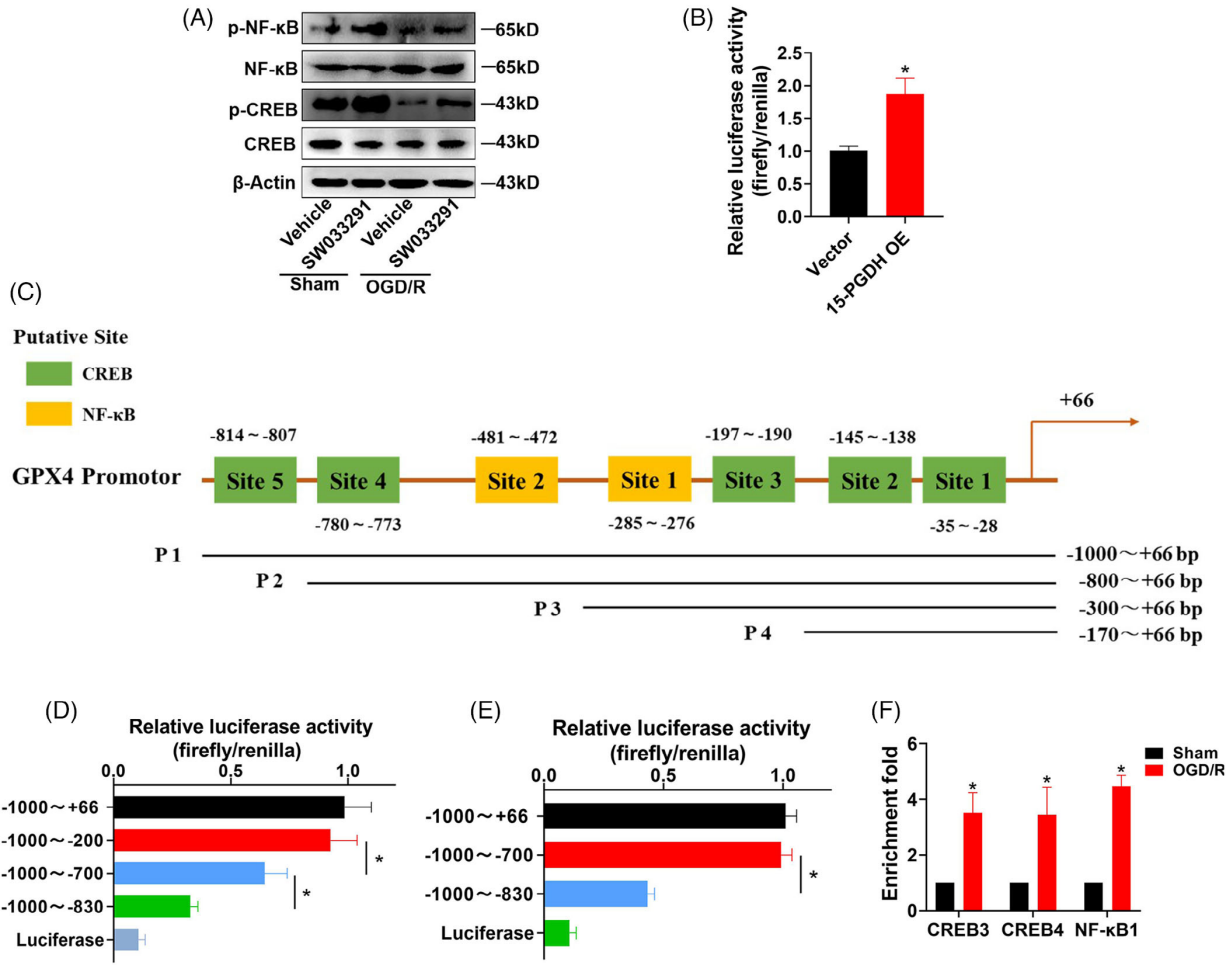
15‐PGDH/PGE2/EP4 transcriptionally regulates GPX4 expression through CREB and NF‐κB. (A) Protein expression of CREB, p‐CREB, NF‐κB, and p‐NF‐κB with or without SW033291 treatment in a OGD/R model. (B) GPX4 promoter activity was analyzed by luciferase assay after truncation of 15‐PGDH overexpression plasmid. (C) Putative CREB‐binding sites and NF‐κB‐binding sites within the 1000 bp region of GPX4 promoter. (D) GPX4 promoter activity was analyzed by luciferase assay after truncation of different CREB putative binding sites. (E) GPX4 promoter activity was analyzed by luciferase assay after truncation of different NF‐κB putative binding sites. (F) CREB and NF‐κB ChIP assay for the GPX4 promoter. Data are shown as the mean ± SD, *n* = 6. *p < 0.05.

Previous results have confirmed that PGE2/EP4 axis and GPX4 mediated the effects of 15‐PGDH on ischemic stroke and ferroptosis, and PGE2/EP4 axis acted upstream of GPX4. As EP4 is potent receptor that activates multiple transcription factors, we hypothesized that EP4 may regulate GPX4 levels at the transcriptional level via some transcription factors. CREB and NF‐κB are representative transcription factors activated by EP4.^25^, ^26^ We focused on the mechanism of CREB and NF‐κB regulated GPX4 transcription. As 15‐PGDH inhibition induced the upregulation of GPX4, we determined whether SW033291 activated CREB and NF‐κB. SW033291 promoted the phosphorylation of CREB and NF‐κB (Figure 6A), indicating that 15‐PGDH inhibition promoted the activation of CREB and NF‐κB. First, 15‐PGDH was confirm to increase the activity of GPX4 promoter (Figure 6B). Next, we predicted 5 putative binding sites for CREB and two putative binding sites for NF‐κB in the 1000 bp of GPX4 promoter from JASPAR database (Figure 6C). We generated four constructs derived from the 1 000 bp promoter fragment of GPX4 according to the location of these putative binding sites of CREB and NF‐κB, including P1 (−1000～+66 bp), P2 (−800～+66 bp), P3 (−300～+66 bp), P4 (−170～+66 bp). We performed dual luciferase reporter assay to determine the key binding sites and found that the sites in −800 to −300 bp and −300 to −170 bp of CREB (Site 3 and 4) and the site in −300 to −170 bp of NF‐κB (Site 1) induced significantly decrease of luciferase activity, indicating that these binding sites were activated to regulate the level of GPX4 (Figures 6D and E). Furthermore, chromatin immunoprecipitation (ChIP) assay revealed that in a OGD/R model, CREB increasingly binded to its putative binding site 3 and 4, and NF‐κB increasingly binded to its putative binding site 2 (Figure 6F). These results suggested that CREB and NF‐κB acted as transcription factors of GPX4.

## 3 DISCUSSION

Though 15‐PGDH is well known as the degrading enzyme of PGE2, recent reports have revealed its function on cell death such as apoptosis and autophagy in cancer and LPS‐induced liver injury or kidney injury.^12^, ^27^, ^28^ However, the role of 15‐PGDH in ferroptosis, a novel form of cell death, has not been proposed. We discover that 15‐PGDH aggravates ischemic stroke in vivo and in vitro, by promoting GPX4‐dependent ferroptosis. Particularly, 15‐PGDH/PGE2/EP4 axis transcriptionally regulates GPX4 expression through CREB and NF‐κB. Hence, the inhibition of 15‐PGDH shows a strong neuroprotective effect on ischemic stroke and might serve as a potential target for ischemic stroke therapies.

To date, the effect of 15‐PGDH on ischemic stroke has not been explored. During a hypoxia animal model, 15‐PGDH was deserved to elevate in internal carotid artery.^29^ While in our previous report, we found 15‐PGDH was decreased in the ischemic hemisphere. We hypothesized that the change pattern of 15‐PGDH may be different in different tissue sites in cerebral ischemia/hypoxic diseases. In this report, we further demonstrated that 15‐PGDH overexpression aggravates ischemic stroke‐induced neurological deficits, cerebral infarction and edema, while 15‐PGDH inhibition shows a neuroprotective effect on ischemic stroke. Similar results were also observed in vitro. We hypothesized that the reduction of 15‐PGDH after MCAO was a kind of self‐protection, but it was not powerful enough to reverse the damage to MCAO. If the level of 15‐PGDH was further reduced artificially, the protective effect of 15‐PGDH inhibition would be stronger. Therefore, although the 15‐PGDH level was decreased by MCAO, further inhibition of 15‐PGDH showed a significant protective effect. We noticed that 15‐PGDH was also reported in other ischemic reperfusion injury (IRI). In renal IRI, inhibition of 15‐PGDH significantly increases renal tissue PGE2 levels and improves vasodilation, increases renal blood flow, in conferring renal protection against renal IRI.^30^ In hepatic IRI, 15‐PGDH inhibition increases PGE2 level and prevents hepatic IRI by inhibiting inflammatory responses and oxidative stress and promoting repair.^31^ Thus, in all IRI that have been studied, the inhibition of 15‐PGDH was revealed to show an effective protection. Since IRI of different organs shares many characteristics, whether inhibition of 15‐PGDH can also protect other IRI, or even all IRI, is a scientific issue of great research value, which may provide effective clues for the treatment of IRI.

The potential roles of 15‐PGDH on cell death were previously revealed in tumor and tissue repair and regeneration. 15‐PGDH is well known as a tumor suppressor, which is observed to be downregulation in colon cancer, pancreatic cancer, and Kras‐driven tumor.^32^, ^33^, ^34^ While restoring the levels of 15‐PGDH in tumor tissue can significantly lead to an inhibition of expansion and an improved prognosis. In aged mice, increased activity of 15‐PGDH is presented in heart, skin, spleen, muscle, and colon, while increasing in aged muscle mass and strength in observed after inhibition of 15‐PGDH.^12^ Autophagy and apoptosis have been shown to be regulated by 15‐PGDH.^12^, ^27^ Here, we demonstrated, for the first time, that 15‐PGDH is a positive regulator of ferroptosis by decreasing the level of GPX4. Ferroptosis is one of the important forms of cell death in ischemic stroke.^17^ GPX4 is an indispensable enzyme that catalyzes GSH antioxidant activity. By reducing GPX4 levels, 15‐PGDH induces enhanced lipid peroxidation in ischemic stroke, leading to a poor prognosis of ischemic stroke. Ferroptosis is also an important form of tumor cell death.^35^ As a classical tumor suppressor, the tumor cell death induced by 15‐PGDH may also be involved in ferroptosis. The impact of 15‐PGDH on cell death deserves further investigation.

Targeting GPX4 is emerging as a promising intervention strategy for ferroptosis. As a selenoprotein, GPX4 is induced by selenium at a transcriptional level via the transcriptional activators TFAP2c and Sp1.^17^ An innovate report reveals that ubiquitination of GPX4 by directly binding to GPX4 protein is an effective strategy to induce ferroptosis.^36^ In this study, we identified 15‐PGDH/PGE2/EP4 pathway as a potential modulator of GPX4. We demonstrated that 15‐PGDH inactivates PGE2/EP4 axis, yet degrades PGE2 and reduces the level of EP4, accompanied by a decrease in GPX4 levels. While inhibition of 15‐PGDH by SW033291 promotes PGE2/EP4 axis, yet inhibits the degradation of PGE2 and increases the level of EP4, and reverts the level of GPX4. In this process, the activity of transcription factors CREB and NF‐κB is increased to transcriptionally upregulate the expression of GPX4, thus ablating ischemic stroke‐induced ferroptosis. Previous studies prefer to reveal the phenomenon that altering the level of GPX4 can interfere with ferroptosis. While rare reports focus on the manner that how the alteration of GPX4 level happens. Here, we identified two transcription factors CREB and NF‐κB of GPX4, indicating the potential transcriptionally regulatory mechanism of GPX4 expression. Maybe this is a significant improvement in the ferroptosis regulatory pathway.

Although PGE2 mediates various effects of 15‐PGDH, direct application of PGE2 is unfortunately impractical. As the estimated half‐life of PGE2 is less than 15 s,^37^ the effects of PGE2 only maintain a short time after administration. Thus, inhibition of 15‐PGDH by SW033291 is a better therapeutic strategy, which has been confirmed in bone homeostasis, hematopoietic regeneration, liver regeneration.^38^, ^39^, ^40^ As a novel small‐molecule inhibitor of 15‐PGDH, SW033291 may act as a protective role on other related diseases, and its application in clinical trials is expected.

Ferroptosis is a complex cell death pathway. More efforts are still needed to explore its mechanism. Currently, known antioxidant pathways against ferroptosis mainly include the GPX4–GSH axis, FSP1–CoQ10 pathway, GCH1–BH4–phospholipid axis, FSP1–ESCRT‐III pathway, and DHODH pathway.^41^, ^42^, ^43^, ^44^, ^45^ In our study, we only explored the regulation of 15‐PGDH on the most classical GPX4–GSH axis. Whether and how 15‐PGDH regulates other antioxidant pathways parallel to GPX4–GSH axis is not determined. This is also the unfinished part of our study and may need to be confirmed by follow‐up studies.

To summarize, our data reveal the impact of 15‐PGDH on ischemic stroke, which is achieved by promoting GPX4‐dependent ferroptosis. Notably, GPX4 is transcriptionally regulated by 15‐PGDH/PGE2/EP4 pathway‐induced activation of CREB and NF‐κB. These findings provide a new regulatory mechanism for GPX4 in ferroptosis and a novel therapeutic target for ischemic stroke.

## 4 METHODS

### 4.1 Patients

Ten tiny brain tissues from patients with brain injury (cancer, trauma, stroke and so on) was obtained during decompression of cranial hypertension, provided by the Department of Neurosurgery, Xiangya Hospital, Central South University. These patients consisted of five males and five females. All participants have provided written informed consent. The study was approved and supervised by the Ethics Committee of the Xiangya Hospital of Central South University, with a human ethics identification number of 2021101119.

### 4.2 Sprague–Dawley rats

Male Sprague–Dawley rats, 150−180 g (for AAV) or 250−300 g (for SW033291) were used. Rats were housed in a 25°C 40−60% humidity‐controlled room, and given free access to food and water. Experiments and animal procedures were approved by the Experimental Animal Center of Central South University (reference 2018sydw0222).

### 4.3 Mice

Male C57BL/6JGPT mice aged 8−12 weeks used in this study were obtained from GemPharmatech Co. Ltd (China). GPX4 CKO mice were generated with GPX4‐flox mice and Fos creERT2 mice. Mice were housed in a 25°C 40−60% humidity‐controlled room, and given free access to food and water. Experiments and animal procedures were approved by the Experimental Animal Center of Central South University (reference 2018sydw0222).

### 4.4 Cell culture

Primary cortical neurons were prepared from embryonic (E16–18) rats under sterile conditions. The pregnant mice were anesthetized with pentobarbital sodium and their embryos were removed laparotomy. The cortex was dissected and homogenized in 0.125% trypsin for 20 min in a 37°C incubator. Cells were plated in neurobasal medium containing 2% B27 and 1% penicillin/streptomycin in 96‐well plates or 25 cm^2^ cell culture flask coated with poly‐d‐lysine. The purity of the primary cortical neurons was validated using NeuN staining. Cells were cultured for 2−3 weeks in a 37°C incubator with 5% CO_2_.

### 4.5 Middle cerebral artery occlusion model

The establishment of MCAO model in rats was performed as previously described to induce the cerebral IRI.^15^ For mice, the MCAO model was processed basically the same as rats. After mice were anesthetized with isoflurane (5% induction, and 2% maintenance), follow‐up surgery was performed under a stereomicroscope. Right carotid artery and vagus nerve were gently dissected and an incision is opened in the external carotid artery. A poly‐l‐lysine‐coated monofilament was inserted from the incision, via the internal carotid artery, blocking the right middle cerebral artery. After 60 min of occlusion, the occluding monofilament was withdrawn to allow for reperfusion.

### 4.6 Oxygen‐glucose deprivation

After washed twice with glucose‐free DMEM, the medium of primary cortical neurons was replaced with DMEM without glucose. Then cells were placed in a 37°C incubator with 95% N_2_ and 5% CO_2_ for 3 h. Finally, the cells were returned to normal neurobasal medium and 37°C incubator with 5% CO_2_.

### 4.7 Lateral ventricle injection

Lateral ventricle injection was performed as previously described.^15^ The dilution of SW033291 (S7900; Selleck) was according to the method presented in 2015^14^ and 0.075 mg/kg was injected 2 h before MCAO model. For AAV (GeneChem Corporation), 150−180 g rats were used and the corresponding stereotaxic coordinates of the injection were lateral −1.2 mm, anteroposterior −0.8 mm and dorsoventral −3.8 mm. One microliter of AAV9 with a titer of 1E+12 v.g./mL was injected into the right lateral ventricle at a flow rate of 0.5 μL /min using a nanomite syringe pump through a Hamilton syringe. The MCAO model was established after 2 weeks.

### 4.8 Plasmid transfection

The 15‐PGDH overexpression plasmid and the plasmids for dual luciferase reporter assay were constructed by GeneChem Corporation. Plasmids were transfected with Lipofectamine 3000 according to the protocol of instruction. After transfection with plasmids for 48 h, the cells were used for experiments.

### 4.9 Neurological deficits analysis

Neurobehavioral scores were performed according to Masao Shmi Izu‐Sasamata's criteria, including : (1) degree of autonomic activity, (2) hemiplegia of left forelimb, (3) unstraight extension of left forelimb during tail lifting, (4) ability to resist lateral thrusts, (5) degree of left inclination, (6) degree of left circle, and (7) reaction to antennae. For the above indicators, 2 means normal, 1 means moderate abnormality and 0 means severe abnormality. The total score ranges from 0 to 14.

### 4.10 Infarct volume measurement

Rats were sacrificed and their brains were collected to freeze at −20°C for 20 min. Frozen brains were sectioned into five or six coronal sections. Brain slices were stained with 2% 2, 3, 5‐triphenyl tetrazolium chloride (TTC; D025‐1; Nanjing Jiancheng) for 30 min from light. The infarct volume was measured and analyzed as previously described.^15^

### 4.11 Brain water content measurement

The whole brain was removed and weighed to obtain wet weight. Then the brain was placed in 110°C over for 24 h and weighed to obtain dry weight. The brain water content was calculated as (wet weight − dry weight)/wet weight × 100%.

### 4.12 Cell viability assay

Cell viability was measured by Cell Counting Kit‐8 (CCK‐8, GK3607; Genview). Cells were plated in 96‐well plates and cultured for 24 h. After treatment, CCK‐8 was added to each well at a concentration of 0.1 μL/μL medium and incubated at 37°C for 1−4 h. After incubation, measured the colorimetric absorbance of each well at 450 nm with a microplate reader and calculated.

### 4.13 PGE2 concentration measurement

The quantitative determination of PGE2 was using PGE2 ELISA Kits (CSB‐E07967r or CSB‐E07966m; CUSABIO) according to manufacturer's instructions. The details of PGE2 concentration measurement have been described previously.^15^ The concentration of PGE2 was eventually normalized to total protein amount of the samples.

### 4.14 Iron concentration measurement

Iron concentration was measured using an Iron Colorimetric Assay Kit (K390; BioVision) according to manufacturer's instruction. The details of iron concentration measurement have been described previously.^15^ The concentration of iron was eventually normalized to total protein amount of the samples.

### 4.15 ROS assay

The ROS was detected using a Reactive Oxygen Species Assay Kit (S0033S; Beyotime Biotechnology) according to manufacturer's instruction. Fluorescence probe DCFH‐DA was directly added to cell samples or was added to tissue samples after preparation of single cell suspension. After incubated at 37°C for 30 min, cells were harvested to detect the fluorescence intensity value at Ex/Em = 488/525 nm.

### 4.16 Lipid peroxidation assay

The MDA was detected using a Lipid Peroxidation MDA Assay Kit (S0131S; Beyotime Biotechnology) according to manufacturer's instruction. Samples were homogenized and centrifuged to obtain the supernatant. The prepared TBA working solution was added to the samples and heated at 100°C for 15 min. Then the samples were centrifuged for supernatant and added to a 96‐well plate to determine the absorbance at 532 nm. The concentration of MDA was eventually normalized to total protein amount of the samples.

### 4.17 GSH concentration measurement

The measurements of GSH and GSSG were using a Glutathione Fluorometric Assay Kit (GSH, GSSG, and Total) (K464; BioVision) according to manufacturer's instruction. The details of GSH and GSSG concentration measurements have been described previously.^15^ The concentrations of GSH and GSSG were eventually normalized to total protein amount of the samples.

### 4.18 HE staining

After rats were sacrificed, the brain samples were separated and fixed for 24 h with 4% paraformaldehyde. After dehydrated and paraffin embedded, the brain samples were sectioned into 4 μm slices. Then the slices were deparaffinized in xylene and hydrated a graded series of alcohol. The sections were stained with hematoxylin and eosin and pictured under an optical microscope.

### 4.19 Western blot

The procedure of Western blot has been described in detail previously.^15^ After extracted and quantified, equal amount of protein was subjected to electrophoresis on an SDS‑PAGE, then transferred to a polyvinylidene fluoride membrane and blocked in 5% nonfat dried milk. The membranes were respectively incubated with primary antibodies against β‑actin (A1978, 1:5000; Sigma), 15‐PGDH (160615, 1:200; Cayman), GPX4 (ab125066, 1:2000, Abcam), NF‐κB (8242, 1:1000; Cell Signaling Technology), p‐NF‐κB (3033, 1:1000; Cell Signaling Technology), CREB (9197, 1:1000; Cell Signaling Technology), and p‐CREB (9198, 1:500; Cell Signaling Technology) at 4°C overnight and incubated with the horseradish peroxidase‑conjugated goat anti‑rabbit IgG (BA1054, 1:5000; Boster Biological Technology) or goat anti‑mouse IgG antibody (BA1050, 1:5000; Boster Biological Technology) for 2 h at room temperature. The bands were detected by ECL‐based chemiluminescence, quantitatively analyzed using ImageJ software and normalized to the β‑actin band density.

### 4.20 Reverse‐transcription quantitative PCR

The procedure of RT‑qPCR has been described in detail previously.^15^ After extracted and quantified, 1 μg RNA was subjected to reverse‐transcription. The amplification of cDNA was 95˚C for 30 s to pre denaturation, next performed over 40 cycles with conditions of 95˚C for 5 s and 60˚C for 34 s with a Two Step SYBR® Prime Script™ RT‑qPCR Kit (Takara). The primer sequences were obtained from Sangon Biotech and presented in Table S1. The relative quantitation of mRNA was analyzed by the 2^−ΔΔCt^ computing method.

### 4.21 Dual luciferase reporter assay

Primary cortical neurons were cotransfected using Lipofectamine 3000 according to the protocol of instruction, with GPX4 promoter truncated plasmids, renilla plasmid, and CREB plasmid or NF‐κB plasmid. After cotransfected 48 h, cells were harvested to detect the luciferase of Firefly and Renilla using a Dual‐Glo Luciferase Reporter Assay System (E1910; Promega).

### 4.22 Chromatin immunoprecipitation

ChIP assay was performed with a Pierce Magnetic ChIP Kit (26157; Pierce) according to the manufacturer's instructions. After crosslinked with 1% formaldehyde, cells were harvested to extract nuclei with Membrane Extraction Buffer and digest with MNase. The samples were sonicated on ice to break nuclear membrane. Added Anti‐RNA Polymerase II Antibody, Normal Rabbit IgG, CREB antibody (1:50; Cell Signaling Technology) or NF‐κB antibody (1:100; Cell Signaling Technology) and incubated at 4°C overnight with mixing. Added magnetic beads to each IP and incubated for 2 h at 4°C with mixing. Collected the beads and eluted, then recovered DNA with a DNA Clean‐Up Column. DNA was used for a qPCR reaction: 95°C for 15 min (step 1), 95°C for 15 s (step 2), 62°C for 1 min (step 3, collect real‐time data) and repeat steps 2 and 3 for 40 cycles. The ChIP primer sequences were obtained from Sangon Biotech and presented in Table S2.

### 4.23 Statistical analysis

All the experiments were repeated independently at least three times. After presented as the mean ± SD, data were analyzed using analysis of variance statistical analysis. The correlation between two indices was performed using Pearson Product‐Moment Correlation. All statistical analysis were performed using GraphPad Prism 8 software. The Adobe Illustrator 2023 was performed to create the Graphical Abstract. Differences were considered statistically significant at *p* < 0.05.

## 5 CONCLUSIONS

In summary, our study indicates that inhibition of 15‐PGDH promotes the activation PGE2/EP4 axis, subsequently transcriptionally upregulates the expression of GPX4 via CREB and NF‐κB, and then protects neurons from ferroptosis and alleviates the ischemic stroke. Therefore, 15‐PGDH may be a potential therapeutic target for ischemic stroke.

## AUTHOR CONTRIBUTIONS

Ying Liu and Jie Zhao designed and directed the project. Yunfei Xu performed the experiments. Yunfei Xu, Kexin Li, Yao Zhao, Lin Zhou, Haoduo Qiao, and Qing Xu analyzed the data. All authors discussed the results and contributed to the final manuscript. Yunfei Xu wrote the manuscript. Ying Liu and Huali Zhang revised the manuscript. Yunfei Xu and Nina He performed the experiments during the process of revision. All authors have read and approved the final manuscript.

## CONFLICT OF INTEREST STATEMENT

The authors declare that they have no conflict of interest.

## ETHICS STATEMENT

This research was approved by the ethics committee of the Xiangya Hospital of Central South University, No: 2021101119. All participants involved in this study provided written informed consent. All procedures performed in studies involving animals were by the ethical standards of the institution or practice at which the studies were conducted by Experimental Animal Center of Central South University (Changsha, China), No: 2018sydw0222.

## Supporting information

Supporting Information

## Data Availability

All data are available in the main text or the supplementary materials.
