# Supplementary material for: Inhibition of 15‐hydroxyprostaglandin dehydrogenase protects neurons from ferroptosis in ischemic stroke
Source: MedComm (2020). 2024 Jan 7;5(1):e452. doi: 10.1002/mco2.452 (PMC10771813; doi:10.1002/mco2.452)
Supplement: Supplementary file 1 — Supporting Information [file MCO2-5-e452-s001.docx]

**Inhibition of 15-** **hydroxyprostaglandin dehydrogenase protects neurons from ferroptosis in** **ischemic stroke**

Yunfei Xu^1,2,3,4,5^, Kexin Li^1,2,3,4^, Yao Zhao^1,2,3,4^, Lin Zhou^1,2,3,4^, Nina He^1,2,3,4^, Haoduo Qiao^1,2,3,4^, Qing Xu^1,2,3,4^, Huali Zhang^1,3,4,*^, Ying Liu^1,3,4,*^, Jie Zhao^2,3,4,*^

^1^Department of Pathophysiology, School of Basic Medical Sciences, Central South University, Changsha 410008, Hunan, China;

^2^Department of Neurosurgery, Xiangya Hospital, Central South University, Changsha 410008, Hunan, China;

^3^Sepsis Translational Medicine Key Lab of Hunan Province, Changsha 410008, Hunan, China;

^4^National Medicine Functional Experimental Teaching Center, Central South University, Changsha, Hunan 410078, P.R. China;

^5^Postdoctoral Research Station of Biology, School of Basic Medical Science, Central South University, Changsha 410008, Hunan, China.

*Correspondence authors:

Dr. Ying Liu,

Department of Pathophysiology, Xiangya School of Medicine, Central South University, Changsha, Hunan, 410078, China;

E-mail: [liu1977ying@126.com](mailto:liu1977ying@126.com)

Dr. Jie Zhao,

Department of Neurosurgery, Xiangya Hospital, Central South University, Changsha 410008, Hunan, China;

1. mail: [steelzj@126.com](mailto:liu1977ying@126.com)

Dr. Huali Zhang,

Department of Pathophysiology, Xiangya School of Medicine, Central South University, Changsha, Hunan, 410078, China;

E-mail: zhanghuali@csu.edu.cn

**Supplemental Material to Fig 1B and 1L (original blots)**


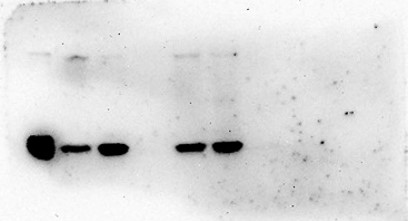

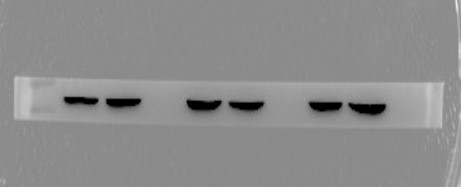


**Fig 1B**

**β-Actin**

**15-PGDH**

**29kD**

**43kD**


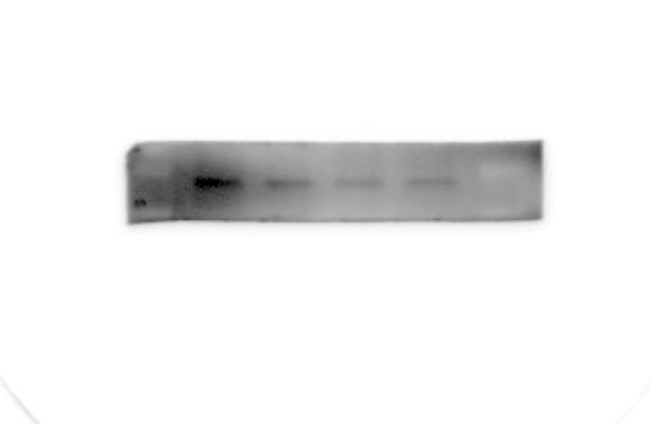

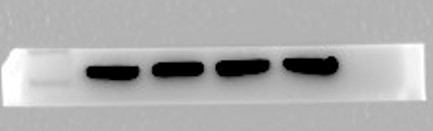


**Fig 1L**

**β-Actin**

**29kD**

**15-PGDH**

**43kD**

**Supplemental Material to Fig 4A and 4D (original blots)**


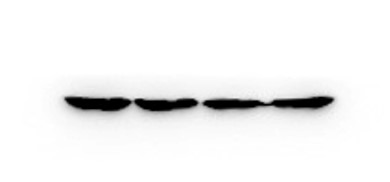

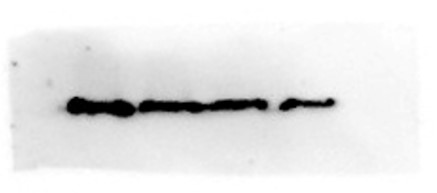


**Fig 4A**

**19kD**

**GPX4**

**43kD**

**β-Actin**

**Supplemental Material to Fig 6C (original blots)**


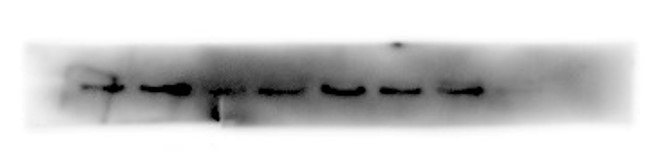

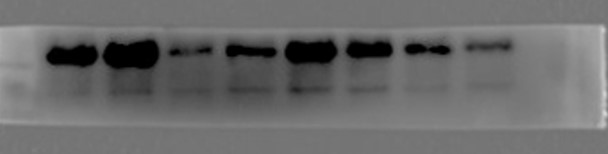

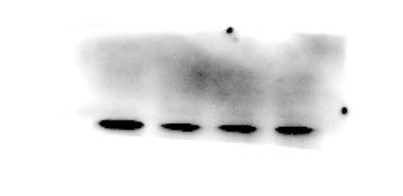

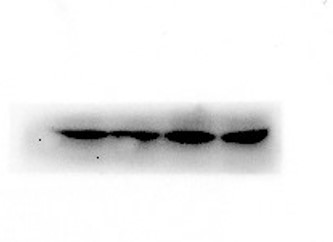

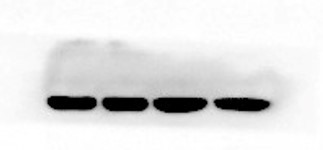


**Fig 6C**

**p-NF-κB**

**65kD**

**43kD**

**p-CREB**

**65kD**

**NF-κB**

**43kD**

**CREB**

**β-Actin**

**43kD**

**Tables**

**Table 1 The primer sequences of RT-qPCR.**

| **Table 1 \| The primer sequences of qRT-PCR.** | | | |
| --- | --- | --- | --- |
| **Primers** | **Species** | **Forward (5^,^–3^,^)** | **Reverse (5^,^–3^,^)** |
| β-actin | Human | CAGATGTGGATCAGCAAGCAGGAG | GTCAAGAAAGGGTGTAACGCAACTAAG |
| 15-PGDH | Human | AAAGCATGGCATAGTTGGATTC | CAAGGATGGCTGTGTTAACAAA |
| β-actin | Rat | TGTCACCAACTGGGACGATA | GGGGTGTTGAAGGTCTCAAA |
| 15-PGDH | Rat | AATGGAGGTGAAGGTGGCAT | CAGTCTCACACCGCTTTTCA |
| SAT1 | Rat | TTTTGGAGAGCACCCCTTCT | CCAAAGCCTCGGTAATCACTC |
| ACSL4 | Rat | AGGATATGATGCCCCTCTTTGT | CATGAATCGGTGTGTCTGGG |
| GPX4 | Rat | TAAGTACAGGGGTTGCGTGT | AGGCCAGGATTCGTAAACCA |
| ALOX15 | Rat | CTTCCTGCCCGCCTGGTATTC | CCGCTTCAAACAGAGTGCCTTTC |
| SLC7A11 | Rat | TCATCATCGGCACCGTCATCG | CTCCACAGGCAGACCAGAACAC |
| EP1 | Rat | TGGTGTTTCATTAGCCTTGGG | GACCTGCGTTCTCTCGGAA |
| EP2 | Rat | ACCGCATACCTTCAGCTGTA | CCTCCGCCATAGAAGTCCTT |
| EP3 | Rat | TGACCATGACAGTGTTCGGA | GCACAGACAGCCACACAC |
| EP4 | Rat | CGCCTACTTCTACAGCCACT | ATGTAAGAGAAGGCGGCGTA |
| β-actin | Mouse | CATTGCTGACAGGATGCAGAAGG | TGCTGGAAGGTGGACAGTGAGG |
| SAT1 | Mouse | CTGAAGGACATAGCATTGTTGG | TTCCATTCTGCTACCAAGAAGT |
| ACSL4 | Mouse | CAATAGAGCAGAGTACCCTGAG | TAGAACCACTGGTGTACATGAC |
| ALOX15 | Mouse | GCAACTGGAAGGATGGCACAATC | TCGCTGGTCTACAGGGAGGTC |
| SLC7A11 | Mouse | TCATGTCCACAAGCACACTCCTC | AGAAGAGCATCACCATCGTCAGAG |
| EP4 | Mouse | CTACTTCTACAGCCACTACGTG | AGAACAGCACGTTAGATGCATA |

**Table 2 The primer sequences of ChIP qPCR.**

| **Table 2 \| The primer sequences of ChIP qPCR.** | | | |
| --- | --- | --- | --- |
| **Primers** | **Species** | **Forward (5^,^–3^,^)** | **Reverse (5^,^–3^,^)** |
| CREB 3 | Rat | TCCGCCAAGCCCTCCTACCACC | TGAATTCTAGCTACATCTGGGG |
| CREB 4 | Rat | CCCTGGCACCCTGCTCCGCTGC | CAACAAACTCCAAGCCCCGCCC |
| NF-κB 1 | Rat | GACGTCGGGAGGTTGCGCATCG | TCTTCTTTTTTTCTCTGAGGAC |
